# Supplementary material for: A randomized, controlled clinical trial demonstrates improved owner-assessed cognitive function in senior dogs receiving a senolytic and NAD+ precursor combination
Source: Sci Rep. 2024 May 29;14:12399. doi: 10.1038/s41598-024-63031-w (PMC11137034; doi:10.1038/s41598-024-63031-w)
Supplement: Supplementary file 4 — Supplementary Information 4. [file 41598_2024_63031_MOESM4_ESM.pdf]

a.

Group Daytime Activity on Weekdays over Time

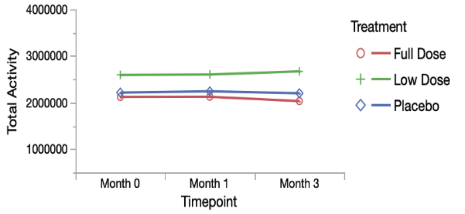

	Month 0	Month 1	Month 3
Full Dose	2121177.33	2124223.24	2032127.19
Low Dose	2594228.34	2602078.25	2669909.66
Placebo	2215007.51	2241396.15	2200677.55

b.

Group Night time Activity on Weekdays over Time

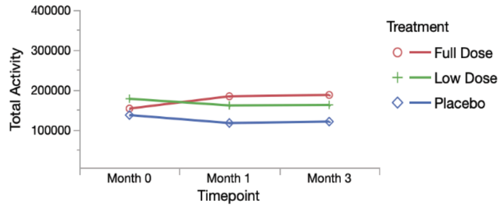

	Month 0	Month 1	Month 3
Full Dose	152509.50	183458.48	186752.90
Low Dose	177181.46	160498.97	161784.77
Placebo	136192.49	116236.34	119815.79

c.

Group Daytime Activity on Weekends over Time

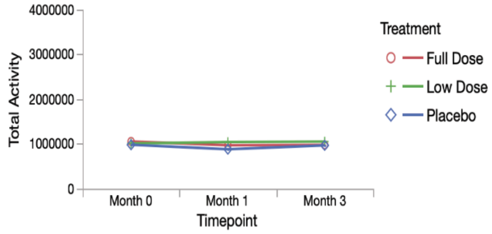

	Month 0	Month 1	Month 3
Full Dose	1056012.75	971746.05	979355.00
Low Dose	1012168.03	1042947.72	1053542.23
Placebo	982300.10	881980.71	968383.09

d.

Group Night time Activity on Weekends over Time

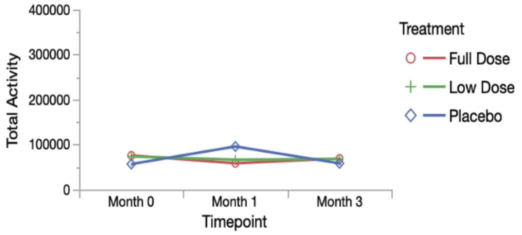

	Month 0	Month 1	Month 3
Full Dose	75983.81	58541.70	69125.24
Low Dose	73766.51	66468.32	68212.73
Placebo	56743.98	95902.81	58369.47

Supplementary Figure S1: Repeated measures analysis of summated activity monitor activity by group.

Repeated measures analysis of summated activity across month 0, 1 and 3. Day (5am-10:59pm) and night (11pm-4:59am) were assessed separately, as were weekdays and weekends. Mean summated activity levels by group were obtained from repeated measures models (MANOVA analysis in JMP) with adjustment for covariates when necessary. The Wilks' lamda value was evaluated, with $p<0.05$ indicating a significant difference between groups. All original activity data is provided in Supplementary Data S3.

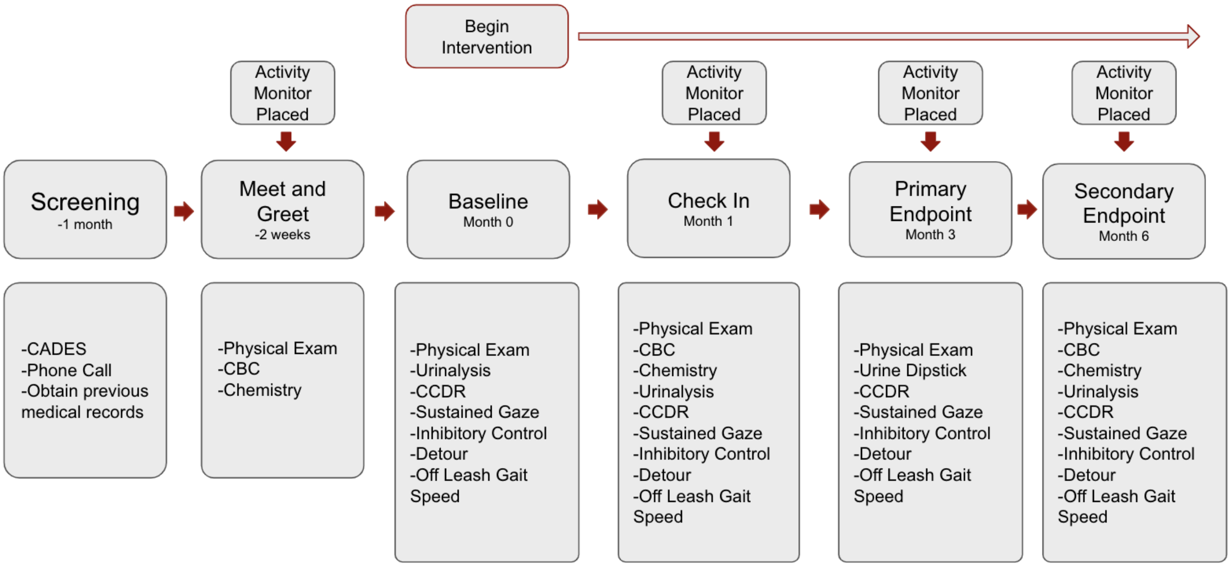

Supplementary Figure S2: Study Timeline. The timeline of all visits in the study are provided along with all assessments performed at each visit. Activity monitors were placed at specific visits in order to obtain two week increments of activity data for each timepoint. Intervention was started the day following an individual's baseline (month zero) visit.
