# Supplementary material for: A randomized, controlled clinical trial demonstrates improved owner-assessed cognitive function in senior dogs receiving a senolytic and NAD+ precursor combination
Source: Sci Rep. 2024 May 29;14:12399. doi: 10.1038/s41598-024-63031-w (PMC11137034; doi:10.1038/s41598-024-63031-w)
Supplement: Supplementary file 5 — Supplementary Information 5. [file 41598_2024_63031_MOESM5_ESM.pdf]

Supplements and Aging - Changes Since Last Visit

Please complete the survey below. Indicate any changes since your pet's last visit. Thank you!

Is this still your current address?<br>[enrollment_arm_1][street_address]<br>[enrollment_arm_1][city_state]<br>[enrollment_arm_1][zipcode]	◯ Yes ◯ No
Please supply your new street address	______________________________
City, State	______________________________
Current zip code	______________________________
Date of address change (approximate)	______________________________
Please describe your home:	◯ House<br>◯ Duplex<br>◯ Townhouse<br>◯ Apartment<br>◯ Other ______
Which type of area do you and your dog live?	◯ Rural (arable farming locally)<br>◯ Suburban<br>◯ City/urban
Is your dog exposed to pesticides/insecticides/herbicides/ fungicides? (these chemicals are often used in the garden to control insects, plants or fungi)	◯ Yes ◯ No
Please specify type	______________________________
Is this still your preferred phone number?<br>[enrollment_arm_1][phone_number]	◯ Yes ◯ No
Please supply your preferred phone number	______________________________<br>(______)
Is this still your preferred email address?<br>[enrollment_arm_1][email1]	◯ Yes ◯ No
Please supply your preferred email address	______________________________
Is [enrollment_arm_1][clinic_name] still [enrollment_arm_1][pet_name]'s primary vet clinic?	◯ Yes ◯ No

Please provide your current primary vet care provider (clinic name and location).	________________________________
Are there any changes regarding the other pets living in the household since [enrollment_arm_1][pet_name]'s last recheck on [visit_month_arm_1][visit_date][previous-instance]? Other pets last reported living in the household: [enrollment_arm_1][current_pets_list]	◯ Yes ◯ No
Please specify changes regarding new pets, or pets that are no longer in the household. Please include pet name, and date entered or left the household. For new pets please include species, breed, age, sex, wt. and relationship to [enrollment_arm_1][pet_name] (ex. gets along, dominates, dislikes, ignores, etc.).	________________________________
Are there any changes to [enrollment_arm_1][pet_name]'s routine medications since [visit_month_arm_1][visit_date][previous-instance]? Current medications listed: [enrollment_arm_1][current_meds_list]	◯ Yes ◯ No
Please indicate medication changes, name, dose, approximate date started or date discontinued.	________________________________
Have you noticed a change in the usage (dose or frequency) of any medications for pain or anxiety that you give on an as-needed basis since your last visit on [visit_month_arm_1][visit_date][previous-instance]? Current as-needed medications listed: [enrollment_arm_1][prn_meds]	◯ Yes ◯ No ◯ Not Applicable
Has this need increased or decreased?	◯ Increased ◯ Decreased
Please specify medication(s) and how the dose and frequency of any as-needed pain or anxiety drug(s) has changed.	________________________________
Has [enrollment_arm_1][pet_name] developed any new medical problems since [visit_month_arm_1][visit_date][previous-instance]? Or have any previous medical problems resolved? Ongoing medical problems last indicated: [enrollment_arm_1][current_medical_list]	◯ Yes ◯ No
Please indicate medical problem, date of onset and/or date resolved.	________________________________
Have you noticed a change in [enrollment_arm_1][pet_name]'s behavior or cognition since your last visit on [visit_month_arm_1][visit_date][previous-instance]?	◯ Worse ◯ The Same ◯ Improved

Have you noticed a change in [enrollment_arm_1][pet_name]'s mobility since your last visit on [visit_month_arm_1][visit_date][previous-instance]?

○ Worse ○ The Same
○ Improved

Compared to your last visit on [visit_month_arm_1][visit_date][previous-instance], [enrollment_arm_1][pet_name]'s level of happiness is:

○ Much worse ○ Slightly worse ○ The same ○ Slightly better ○ Much better

---

Have you noticed any of the following signs since [enrollment_arm_1][pet_name]'s last visit on [visit_month_arm_1][visit_date][previous-instance]? (check all that apply)

☐ vomiting
☐ diarrhea
☐ loss of appetite
☐ itching
☐ ataxia/wobbly gait
☐ increased water intake
☐ increased anxiety
☐ changes in behavior, describe ______
☐ other, describe ______
☐ none of the above

Canine Cognitive Dysfunction Rating

Please indicate the frequency of your dog's behaviors.

	Never	Once a month	Once a week	Once a day	> Once a day
1. How often does your dog pace up and down, walk in circles and/or wander with no direction?	○	○	○	○	○
2. How often does your dog stare blankly at the walls or floor?	○	○	○	○	○
3. How often does your dog get stuck behind objects and is unable to get around them?	○	○	○	○	○
4. How often does your dog fail to recognize people or pets?	○	○	○	○	○
5. How often does your dog walk into walls or doors?	○	○	○	○	○
6. How often does your dog walk away while, or avoid, being petted?	○	○	○	○	○

	Never	1-30% times	31-60% times	61-99% times	Always
7. How often does your dog have difficulty finding food dropped on the floor?	○	○	○	○	○

Please note the answer choices reflect the CHANGE in your dog's behavior over the past 6 months. If your dog has never exhibited the behavior, please select "the same".

	Much less	Slightly less	The same	Slightly more	Much more
8. Compared with 6 months ago, does your dog now pace up and down, walk in circles and/or wander with no direction or purpose?	○	○	○	○	○
9. Compared with 6 months ago, does your dog now stare blankly at the walls or floor?	○	○	○	○	○
10. Compared with 6 months ago, does your dog urinate or defecate in an area it has previously kept clean?	○	○	○	○	○
11. Compared with 6 months ago, does your dog have difficulty finding food dropped on the floor?	○	○	○	○	○

12. Compared with 6 months ago, does your dog fail to recognize familiar people or pets?	○	○	○	○	○
	Much less	Slightly less	The same	Slightly more	Much more
13. Compared with 6 months ago, is the amount of time your dog spends active?	○	○	○	○	○

Total CCDR score ______________________________

Frailty Phenotype Questionnaire and Scoring Guide for Population 2

1. Nutrition:

- Body Condition Score (1 - 9)

  ▢ 1 ▢ 2 ▢ 3 ▢ 4 ▢ 5 ▢ 6 ▢ 7 ▢ 8 ▢ 9

- My dog now:

  ▢ Has an increased appetite compared to 6 months ago

  ▢ Eats normally (i.e. eats regular food, and finishes in the same amount of time)

  ▢ Will still eat their regular food but takes longer to finish

  ▢ Does not want to eat their regular food but will still eat treats or people food

  ▢ Acts interested in food but walks away from the bowl without eating

  ▢ Cannot be enticed to eat no matter what you offer

2. Social Activity:

- Over the past week, how many days has your dog been playful?

  (No days) ▢ 0 ▢ 1 ▢ 2 ▢ 3 ▢ 4 ▢ 5 ▢ 6 ▢ 7 (Everyday)

3. Exhaustion:

- How often does your dog rest (stop, sit, or lie down) during exercise?

  ▢ Never

  ▢ Hardly ever

  ▢ Occasionally

  ▢ Frequently

  ▢ Very frequently

4. Mobility:

- On average, how frequently does your dog display weakness or lameness in his/her limbs when walking?

  Weakness/ Lameness is defined as reduced strength in a limb. This can appear as limping (placing less weight on one leg compared to another), increased effort moving the limbs, scuffing of the paws, short strides, or falling.

  - ▢ Not at all
  - ▢ Less than 10% of the time
  - ▢ 10 - 50% of the time
  - ▢ More than 50% of the time

- On average, how frequently does your dog display stiffness in his/her limbs when walking?

  Stiffness is defined as reduced flexing of the joints. This can present as reduced bending of the limbs, shorter strides, or the dog appearing to be "straight-legged".

  - ▢ Not at all
  - ▢ Less than 10% of the time
  - ▢ 10 - 50% of the time
  - ▢ More than 50% of the time

- Does your dog ever make mistakes while walking?

  Mistakes may include scuffing of the paws, stumbling, swinging the limbs abnormally outward or inward such that they cross, a lack of coordination between the limbs, and/or skipping steps.

  ▢ No ▢ Yes

5. Muscle Condition:

- Forelimb Muscle Condition Score (0 - 3)

  ▢ 0 ▢ 1 ▢ 2 ▢ 3

- Hindlimb Muscle Condition Score (0 - 3)

  ▢ 0 ▢ 1 ▢ 2 ▢ 3

- Epaxial Muscle Condition Score (0 - 3)

  ▢ 0 ▢ 1 ▢ 2 ▢ 3

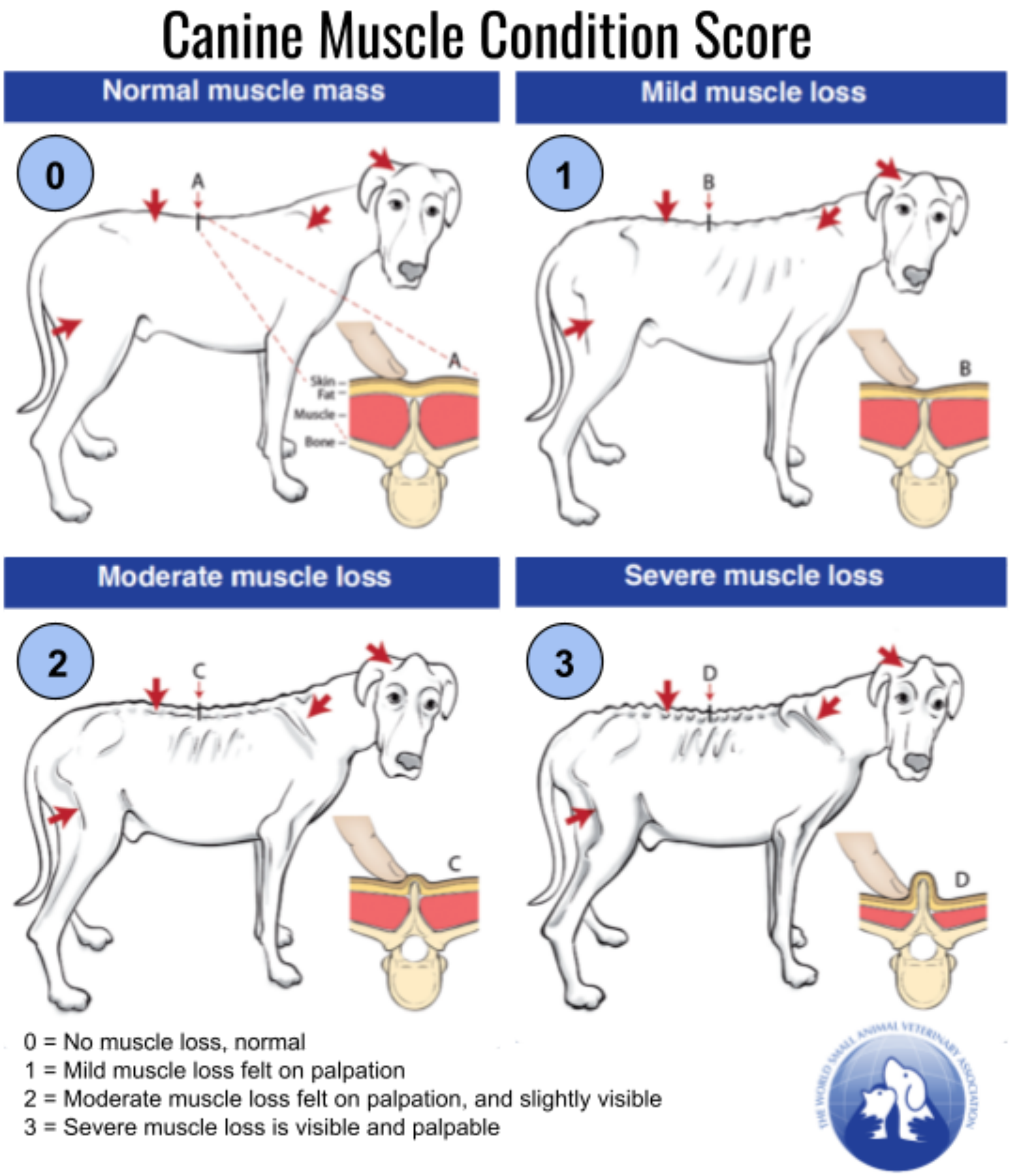

Frailty Phenotype Scoring

Use the following key to complete the summary section and determine the total impaired domains

---

1. Nutrition:

- Body Condition Score (1 - 9)

  ▢ 1 ▢ 2 ▢ 3 ▢ 4 ▢ 5 ▢ 6 ▢ 7 ▢ 8 ▢ 9

- My dog now:

  ▢ Has an increased appetite compared to 6 months ago

  ▢ Eats normally (i.e. eats regular food, and finishes in the same amount of time)

  ▢ Will still eat their regular food but takes longer to finish

  ▢ Does not want to eat their regular food but will still eat treats or people food

  ▢ Acts interested in food but walks away from the bowl without eating

  ▢ Cannot be enticed to eat no matter what you offer

---

2. Social Activity:

- Over the past week, how many days has your dog been playful?

  (No days) ▢ 0 ▢ 1 ▢ 2 ▢ 3 ▢ 4 ▢ 5 ▢ 6 ▢ 7 (Everyday)

---

3. Exhaustion:

- How often does your dog rest (stop, sit, or lie down) during exercise?

  ▢ Never

  ▢ Hardly ever

  ▢ Occasionally

  ▢ Frequently

  ▢ Very frequently

---

5. Muscle Condition:

- Forelimb Muscle Condition Score (0 - 3)

  ▢ 0 ▢ 1 ▢ 2 ▢ 3

- Hindlimb Muscle Condition Score (0 - 3)

  ▢ 0 ▢ 1 ▢ 2 ▢ 3

- Epaxial Muscle Condition Score (0 - 3)

  ▢ 0 ▢ 1 ▢ 2 ▢ 3

Sum of MCS:

▢ 0 ▢ 1 ▢ 2 ▢ 3 ▢ 4 ▢ 5 ▢ 6 ▢ 7 ▢ 8 ▢ 9

<u>Summary of All Frailty Domains</u>

For each domain, if any answer fell within the red zones, that domain receives a score of 1
If no answers for a domain fell within the red zones, then that domain receives a score of 0

Sum the five domains to determine the **<u>Total Impaired Domains</u>**

NUTRITION	SOCIAL ACTIVITY	EXHAUSTION	MOBILITY	MUSCLE CONDITION

**Total Impaired Domains**	

Frailty Interpretation

Non-Frail	Frail
Total Impaired Domains of 0, 1, or 2	Total Impaired Domains of 3, 4, or 5

4. Mobility:

- On average, how frequently does your dog display weakness or lameness in his/her limbs when walking?

  Weakness/ Lameness is defined as reduced strength in a limb. This can appear as limping (placing less weight on one leg compared to another), increased effort moving the limbs, scuffing of the paws, short strides, or falling.

  - ▢ Not at all (0 points)
  - ▢ Less than 10% of the time (1 point)
  - ▢ 10 - 50% of the time (2 points)
  - ▢ More than 50% of the time (3 points)

- On average, how frequently does your dog display stiffness in his/her limbs when walking?

  Stiffness is defined as reduced flexing of the joints. This can present as reduced bending of the limbs, shorter strides, or the dog appearing to be "straight-legged".

  - ▢ Not at all (0 points)
  - ▢ Less than 10% of the time (1 point)
  - ▢ 10 - 50% of the time (2 points)
  - ▢ More than 50% of the time (3 points)

- Does your dog ever make mistakes while walking?

  Mistakes may include scuffing of the paws, stumbling, swinging the limbs abnormally outward or inward such that they cross, a lack of coordination between the limbs, and/or skipping steps.

  ▢ No (0 points) ▢ Yes (1 point)

Sum of Mobility Scores:

▢ 0 ▢ 1 ▢ 2 ▢ 3 ▢ 4 ▢ 5 ▢ 6 ▢ 7

The effect of combined supplementation on activity and attention in aging dogs

Patient label

Study ID: _________ Date:_____________ Visit month #:___________ Estimated Life stage:_____________

Weight (kg): __________ Temp: _________ Pulse: _________ Respiration: _________ MM/CRT: ________

Heart murmur (1-6): _________ BCS (1-9):_______

	Normal	Abnormal	Describe
EENT/oral			
CVR			
GIT/ABD			
MSK			
LYMPH			
DERM			
GU			
Ortho			

EXAMINATION NOTES:

____

____

UPDATES SINCE LAST VISIT

____

____

____

Neurologic exam

Patient label

Date:______________ Visit month #:________

1. Mental Status:_______________ Alert Depressed Disoriented Stuporous Comatose Other
2. Posture:______________________ Normal Head Tilt Head Turn Other (describe)
3. Gait:_________________________ Normal Ataxia Paresis Plegia Lame Circling
4. Palpation: (0 = normal, 1 = mild, 2 = moderate, 3 = severe)

a.Neck pain: ____________ b.TL pain: _____________ c. LS pain: ___________ d. Head pain_________

5. Postural reactions: 0(absent), 1(reduced), 2(normal), 3(increased), 4(clonus)

	LF	RF	LH	RH
CP				
Hopping				

6. Cranial nerves: 0(absent), 1(reduced), 2(normal), 3(increased), 4(clonus)

	L	R
Menace (II, VII)		
Palpebral (V, VII)		
Oculovestibular reflex (III, VI, VIII)		
Gag reflex/Jaw tone (IX, X, V)		
Eye Position/Strabismus (III – VL, VI – medial)		
Facial muscles – eyelid, ear, lip, nose- (VII)		
Masticatory muscles – (V)		
Facial Sensation – (V)		
Nystagmus – (VIII)		

7. Spinal reflexes: 0(absent), 1(reduced), 2(normal), 3(increased), 4(clonus)

	LH	RH	LF	RF
Patellar reflex				
Cranial Tibial reflex				
Sciatic reflex				
Flexor Withdrawal				
Panniculus				
Deep Pain				
Perineal Reflex				

Joint Evaluation

Patient label

Study ID:________ Date:_____________ Visit month #:________

Forelimb	ROM		Pain		Crepitus		Effusion		Thickening	
	L	R	L	R	L	R	L	R	L	R
Manus										
Carpus										
Elbow										
Shoulder										

Hindlimb	ROM		Pain		Crepitus		Effusion		Thickening	
	L	R	L	R	L	R	L	R	L	R
Pes										
Tarsus										
Stifle										
Hip										

Range of Motion:
0: normal; **1**: mild-moderate decreased; **2**: severely decreased

Pain based on manipulation:
0: Does not notice; **1**: Orients to site, does not resist or mild resistance (mild); **2**: Orients to site, slight objection to manipulation (moderate); **3**: Withdraws from manipulation, may vocalize, may turn to guard area (significant); **4**: Tries to escape/prevent manipulation, may bite or show aggression (severe)

Crepitus:
0: none, no crunching; **1**: mild, only feel crunching sometimes **2**: moderate, crunching felt always, may be painful;
3: severe, can feel and hear crunching, may be painful

Effusion:
0: none, no fluid pocket felt; **1**: mild, small fluid pocket felt only on palpation; **2**: moderate, prominent on palpation; **3**: severe, may see visible fluid pocket

Thickening:
0: none, can feel all anatomic structures easily; **1**: mild, less defined anatomic structures; **2**: moderate, can slightly define anatomic structures;
3: severe, can no longer feel anatomic structures
