# Supplementary material for: A randomized, controlled clinical trial demonstrates improved owner-assessed cognitive function in senior dogs receiving a senolytic and NAD+ precursor combination
Source: Sci Rep. 2024 May 29;14:12399. doi: 10.1038/s41598-024-63031-w (PMC11137034; doi:10.1038/s41598-024-63031-w)
Supplement: Supplementary file 6 — Supplementary Information 6. [file 41598_2024_63031_MOESM6_ESM.pdf]

	Time Period	Placebo (n= 20, 18)	Low Dose (n= 21, 17)	Full Dose (n= 18, 18)	p value	Fisher's Exact Test
Changes to Household	Month 0 to Month 3	Yes: 7<br>No: 13	Yes: 2<br>No: 19	Yes: 4<br>No: 14	0.13	0.15
	Month 3 to Month 6	Yes: 3<br>No: 15	Yes: 1<br>No: 16	Yes: 4<br>No: 14	0.35	0.50
Changes to Medication	Month 0 to Month 3	Yes: 6<br>No: 14	Yes: 8<br>No: 13	Yes: 3<br>No: 15	0.24	
	Month 3 to Month 6	Yes: 4<br>No: 15	Yes: 4<br>No: 14	Yes: 3<br>No: 17	0.32	

Supplementary Table S1: Household and medication changes in participants over the course of the study by group.

	Placebo (n=17)	Low Dose (n=17)	Full Dose (n=17)	p value
CCDR Score	0<br>(-11 - 7)	0<br>(-11 - 14)	1<br>(-6 - 26)	0.44
Frailty Score	0<br>(-2 - 2)	0<br>(-1 - 2)	0<br>(-1 - 3)	0.80
Cylinder Task (Inhibitory Control) (%)	0<br>(-25 - 25)	0<br>(-50 - 37.5)	0<br>(-75 - 37.5)	0.33
Detour (%)	0<br>(-75 - 37.5)	0<br>(-87.5 - 37.5)	0<br>(-25 - 37.5)	0.28
Sustained Gaze (sec)	-4.24<br>(-42.53 - 22.37)	2.18<br>(-25.82 - 17.34)	-1.11<br>(-20.25 - 29.28)	0.33
Off-Leash Gait Speed (m/s)	0.02<br>(-0.83 - 0.99)	-0.08<br>(-0.68 - 0.78)	0.05<br>(-0.66 - 0.43)	0.64

Supplementary Table S2: Median (Range) change in outcome measures (Month 6 - Month 3) by group.

			Cumulative Change in Activity	p value
Weekday Activity	Daytime	Placebo (n=16)	124158 (-954337 - 632044)	0.89
		Low Dose (n=16)	142019 (-344941 - 1253340)	
		Full Dose (n=17)	-25522 (-805479 - 753977)	
	Nighttime	Placebo (n=16)	10092 (-25852 - 125521)	0.10
		Low Dose (n=16)	4609 (-98981 - 190055)	
		Full Dose (n=17)	-17168 (-139014 - 102041)	
Weekend Activity	Daytime	Placebo (n=16)	139652 (-589871 - 962594)	0.60
		Low Dose (n=16)	113584 (-395776 - 548630)	
		Full Dose (n=17)	7127 (-597520 - 398160)	
	Nighttime	Placebo (n=16)	-1871.64 (-75114 - 78950)	0.83
		Low Dose (n=16)	1822 (-65863 - 128574.97)	
		Full Dose (n=17)	7243 (-129861 - 167042)	

Supplementary Table S3: Median (range) cumulative change in activity (month 6 - month 3) by group.

Adverse Events by System, Severity and Treatment Group

			Placebo			Low Dose			Full Dose		
			Month 1	Month 3	Month 6	Month 1	Month 3	Month 6	Month 1	Month 3	Month 6
**System Category**	**Adverse Event**	**VCOG Grade**									
**Allergic/Immunologic Event**	Anaphylaxis	2									n=1
**Body Cavity**	Ascites	4			n=1						
**Cardiac**	Arrhythmia	1	n=1								
	Hypertension	2					n=1				
		1				n=1					
**Dermatologic/Skin**	Acute Dermatitis	2		n=1							
	Atopic Dermatitis	2							n=1	n=1	
	Hygroma	1				n=1					
	Preputial infection	2				n=1					
	Pruritus	1	n=1	n=1		n=1	n=4	n=3	n=3	n=2	n=2
**Dental**	Tooth Decay	3		n=1							
	Tooth Root abscess	3	n=1								
**Ear Disorders**	Ear infection	2		n=1			n=1				
**Gastrointestinal**	Anal Gland Infection	2				n=1					
	Acid Reflux	1					n=1				
	Appetite-Increased	1	n=1	n=2	n=1	n=4	n=2	n=3	n=1		n=1
		2	n=1		n=1		n=1				
	Appetite-Decreased	1		n=1	n=1	n=2	n=3	n=1	n=3	n=2	n=2
	Constipation	1					n=1				
	Diarrhea	2				n=1	n=2				
		1	n=3	n=1		n=3	n=1	n=4	n=3	n=3	n=2
	Fecal Incontinence	5						n=1			
		2			n=1						
		1					n=1				
	Gastric Dilation-Volvulus	5								n=1	
	Gastroenteritis	2									n=1
	Irritable Bowel Disease flare-up	2	n=1	n=1							
	Vomiting	2			n=1	n=1	n=1				
		1	n=3		n=1	n=1	n=1	n=1	n=3	n=1	n=2

**Hepatobiliary**	Hepatomegaly	3			n=1						
	Non-obstructive Gallbladder Stone	1				n=1					
**Musculoskeletal**	Hindlimb Weakness	3									n=1
		2								n=1	
	Lameness	2	n=1	n=1						n=1	
		1				n=2					
**Neoplasm**	Adrenal Mass	2			n=1						
	Cardiac Mass	5						n=1			
	Lymphoma	5						n=1			
	Oral Mucosal Mass	2				n=1					
	Splenic Mass	5		n=1							
	Soft Tissue Sarcoma	3					n=1				
	Tail Mass	3						n=1			
	Thoracic Mass	1			n=1						
**Neurology**	Acute Neck Pain	2						n=1			
		1							n=1		
	Anxiety	1	n=1	n=3		n=2	n=1	n=1	n=1	n=2	n=3
	Ataxia/Wobbly gait	1	n=2	n=3	n=4	n=2	n=2	n=2	n=3		n=4
	Depression	1				n=1					
	Horner's Syndrome	1		n=1							
	Cauda Equina Syndrome	3			n=1						
	Seizure	2					n=1	n=1			
		1				n=1					
	Vestibular Episode	2				n=1				n=1	
		1						n=1			n=1
**Ocular**	Corneal Ulcer	2	n=1								n=1
	Refractory Ulcer	2			n=1						
	Ocular Discharge	1				n=1					n=1
**Renal/Genito**	Bladder Stones	2						n=1			
	Polyuria/ Polydipsia	2		n=2	n=1	n=1					n=2
		1	n=5	n=5	n=5	n=3	n=3	n=3		n=1	
	Urinary Incontinence	5						n=1			
		1				n=1	n=1				
	Inappropriate Urination	1		n=2	n=3	n=2	n=2	n=1	n=2	n=1	n=3
**Resp/Pulm**	Aspiration Pneumonia	2						n=1			
	Coughing	1				n=1	n=1			n=1	

	Collapsed Lung/ Pneumonia	5			n=1						
	Increased Respiratory Effort	1	n=1								
	Panting	1				n=2		n=1			n=1
	Tracheal Collapse	2						n=1			

Supplementary Table S4: Adverse Events by Group

Lab Work Changes by System, Severity and Treatment Group

			Placebo			Half Dose			Full Dose		
			Month 1	Month 3	Month 6	Month 1	Month 3	Month 6	Month 1	Month 3	Month 6
**System Category**	**Adverse Event**	**VCOG Grade**									
Blood/Bone Marrow	Anemia	1	n=2		n=5	n=6		n=4	n=1		n=3
	Lymphopenia	1	n=4		n=1	n=5		n=4	n=2		n=5
	Monocytosis	1	n=2			n=1			n=1		
	Monocytopenia	`							n=1		
	Plasma Protein-Decreased	1	n=1		n=2				n=1		n=1
	Plasma Protein-Increased	1	n=4		n=1	n=3		n=1	n=1		
	Stress Leukogram	1			n=1	n=1		n=1			
	Thrombocytosis	1			n=2						
	Thrombocytopenia	1							n=1		n=1
Metabolic/ Laboratory	Albumin-Increased	1						n=1			
	Amylase-Increased	1			n=2			n=1			n=1
	Anion Gap-Decreased	1									n=1
	ALP-Increased	1	n=1		n=2	n=3		n=3			n=1
	ALT-Increased	3									n=1
		1			n=1			n=3			n=2
	AST-Increased	1			n=1	n=2		n=1	n=2		n=1
	Bicarbonate-Decreased	1	n=1		n=1						
	Bicarbonate-Increased	1	n=1						n=1		
	BUN-Decreased	1	n=1		n=1						
	BUN-Increased	2						n=1			n=1
		1	n=1			n=1		n=1	n=3		n=1
	Creatinine-Decreased	1			n=1	n=1					
	Creatinine-Increased	1				n=2		n=1	n=1		n=2
	Creatine Kinase-Increased	1	n=2			n=4		n=1			
	GGT-Increased	1			n=1	n=1		n=1			n=3
	Globulins-Decreased	1	n=1		n=2						n=1
	Hyperkalemia	1	n=3		n=1	n=1					n=1
	Hypercalcemia	1						n=2	n=1		n=4
	Hyperchloremia	1			n=1						

	Hypermagnesemia	1							n=1		n=2
	Hyperphosphatemia	1			n=1				n=1		n=1
	Hypocalcemia	1			n=1	n=1					
	Hypochloremia	1			n=1	n=1			n=1		
	Hypoglycemia	1									n=1
	Hypomagnesemia	1	n=1		n=1	n=1		n=1	n=1		
	Hyponatremia	1	n=2		n=5	n=1		n=1	n=3		n=3
	Hypophosphatemia	1				n=2		n=2	n=1		n=1
	Lipase-Increased	1	n=1		n=3	n=1					n=2
	Total Protein-Decreased	1	n=1		n=1	n=1					
**Renal/ Genitourinary**	Bacteriuria	1	n=1		n=3	n=1	n=1	n=1	n=4	n=1	n=2
	Bilirubinuria	1			n=3	n=1	n=1		n=1	n=1	n=1
	Hematuria	1	n=3	n=1	n=5	n=5	n=2	n=4	n=4		n=2
	Isosthenuria	1			n=1	n=1					n=1
	Ketonuria	1	n=3		n=6	n=3	n=1	n=3	n=3		n=1
	Proteinuria	1	n=1			n=5	n=1	n=3	n=1		n=1

Supplementary Table S5: Changes in Patient Lab Work by Group
